# A new species of *Sphaerius* Waltl from China (Coleoptera, Myxophaga, Sphaeriusidae)

**DOI:** 10.3897/zookeys.808.30600

**Published:** 2018-12-18

**Authors:** Zu-long Liang, Fenglong Jia

**Affiliations:** 1 Institute of Entomology, Life Science School, Sun Yat-sen University, Guangzhou, 510275, Guangdong, China Sun Yat-sen University Guangdong China

**Keywords:** China, Coleoptera, new species, Oriental Region, *
Sphaerius
*, Sphaeriusidae

## Abstract

A new species, *Sphaeriusminutus***sp. n.**, is described and illustrated from Jinggangshan Mts., Jiangxi Province, China. It is the first species of this family described from East Asia. This species lives under wet stones at the edge of rivers.

## Introduction

Sphaeriusidae are a group of tiny beetles, usually 0.5–1.3 mm in length, most of which are semi-aquatic, living in moist substrates by stream or river banks, but some species are strictly terrestrial (Löbl 1995, Endrödy-Younga 1997, Beutel and Arce-Pérez 2005). The family contains a single genus, *Sphaerius* Waltl, 1938 with 22 valid species, distributed in all continents expect Antarctica (Reichardt 1973, Löbl 1995, Lawrence and Ślipiński 2013). Among all faunal regions, the Oriental Region has the largest number of species. Reichardt (1973) recorded 18 known species of Sphaeriusidae, eight of which were from Southeast Asia. Subsequently four more terrestrial species were reported from South Asia (Löbl 1995). However, none of the species have ever been described from East Asia although this family has been reported from China once by Hall (2003) but without any detailed information. This family has also been reported from several places in Japan but no specimen was identified to species level (Kamezawa and Matsubara 2012). Other than that, there are no other records of this family from China or other East Asian countries. Based on the specimens collected from Jiangxi, China, a new species of *Sphaerius* is described. It is the first species of *Sphaerius* described from East Asia.

## Materials and methods

Specimens of the new species were dissected, and the genitalia were placed in a drop of glycerol on glass slides. After photography, the genitalia were transferred to a plastic plate attached to the respective specimen. SEM photographs were taken with a Phenom Prox scanning electronic microscope, and photographs of habitus and male genitalia were taken with a Zeiss SteREO Discovery V20 Microscope and a Zeiss Axioskop 40 Microscope respectively. Illustrations of elytron and male genitalia were drawn with Adobe Illustrator CS6 based on the material and photographs.

One paratype was deposited in National Museum, Prague, Czech Republic (**NM**), and the remaining material examined is at the Entomological Collection of Sun Yat-sen University, Guangzhou, China (**SYSU**).

## Results

### Sphaerius minutus sp. n.

Taxon classificationAnimaliaColeopteraSphaeriusidae

http://zoobank.org/1D941BED-6051-427B-8080-B1D415975C64

Figs 1–9 , 10–18

#### Type material.

**Holotype**: male: China, Jiangxi Province, Jinggangshan Mountain, 1.3 km southwest of Xiping County, 26°33'4"N, 114°12'2"E, 850 m, at the edge of a stream, beneath a stone, caught, 24 June 2011, Fenglong Jia leg. **Paratype**: five specimens: same data as holotype.

#### Diagnosis.

Length 0.9–1.0 mm, broadly oval, strongly convex in form. Dorsal surface smooth and shiny, without any punctation or striae. Each elytron with 8–12 conical and rough asperities. Antennae with last four antennomeres slightly clubbed, but not as strongly dilated as in other species of the genus. Prosternal process T-shaped, strongly elevated, with anterior margin straight and lateral margin smoothly concave. Pro- and mesotibiae each bear a ventral disc at the apex. Median lobe of the aedeagus long and laterally compressed, apex distinctly dilated, apical angle rounded, never prominent; left paramere digitus-shaped, half the length of the median lobe; right paramere petaliform.

#### Description.

**Figures 1–9.**
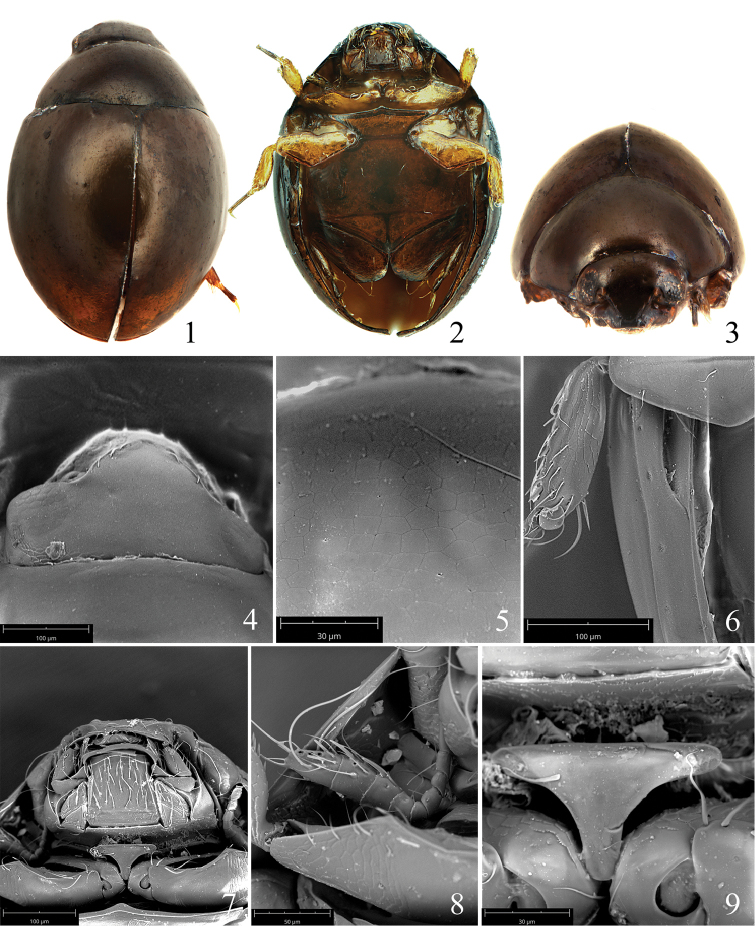
*Sphaeriusminutus* sp. n. **1–3** habitus (**1** dorsal view **2** ventral view **3** frontal view) **4** dorsal view of head **5** micro-reticulation on pronotum **6** epipleuron **7** ventral view of head **8** antenna **9** prosternal process.

Body widely oval, strongly convex in form. 0.9–1.0 mm in length, ca. 1.5× as long as wide, widest in the middle. Dorsal surface uniformly brown to dark brown, smooth and shining, without any punctation (Fig. 1). Ventral surface reddish brown, with front legs and mid legs yellow (Fig. 2). Micro-reticulation distinct on head and pronotum at magnification of more than × 1000, but fainter on elytra. The meshes of reticulation pentagonal or hexagonal (Fig. 5).

*Head.* Short and broad. Frons attenuated anteriorly, with several setae along lateral margin. Clypeus short and broad, with anterior margin straight, fronto-clypeal suture indistinct. Compound eyes developed, strongly protruding (Figs 3, 4). Mentum flat, shovel-like, slightly attenuated anteriorly, anterior margin slightly rounded; lateral margin with two longitudinal ridges on each side (Fig. 7). Antennae inserted near eyes, extending well beyond the posterior margin of prosternum, 11–segmented; antennomere 3 long, about as long as the following 4 antennomeres combined; last 4 antennomeres slightly clubbed, but not dilated as strongly as in other species, at most twice as wide as previous antennomeres, with rough and long setae at apex (Fig. 8).

*Pronotum.* Attenuated anteriorly, ca. 0.35× as long as wide, lateral margin smoothly rounded; anterior angle produced and acute, posterior angle rectangular, slightly rounded; posterior margin slightly narrower than base of elytra. Scutellum almost equilaterally triangular, small and flat, with length a bit narrower than wide (Fig. 1).

*Elytra.* Smooth and shining, without any punctation or striae, lateral margin smoothly rounded (Fig. 5). Elytron with 8–12 conical and rough asperities arranged as in Fig. 15, some of which are obsolete or absent in some specimens. Epipleuron developed, constricted posteriorly, with a deep and broad furrow along basal third; outer edge with several round depressions composed of a cluster of micro-holes (Figs 6, 11).

*Ventral surface.* Prosternal process T-shaped, strongly elevated, ca. 0.6× as long as wide, extending to about half of procoxae; anterior margin straight, while lateral margin smoothly concave (Fig. 9). Mesoventrite short and broad, largely elevated at middle, constricted posteriorly, reaching the middle of mesocoxae, fused with the much larger metaventrite; metaventrite ca. 2.5× as long as mesoventrite; meso- and metaventrite smooth and flat, bearing very sparse bristles (Fig. 2).

**Figures 10–18.**
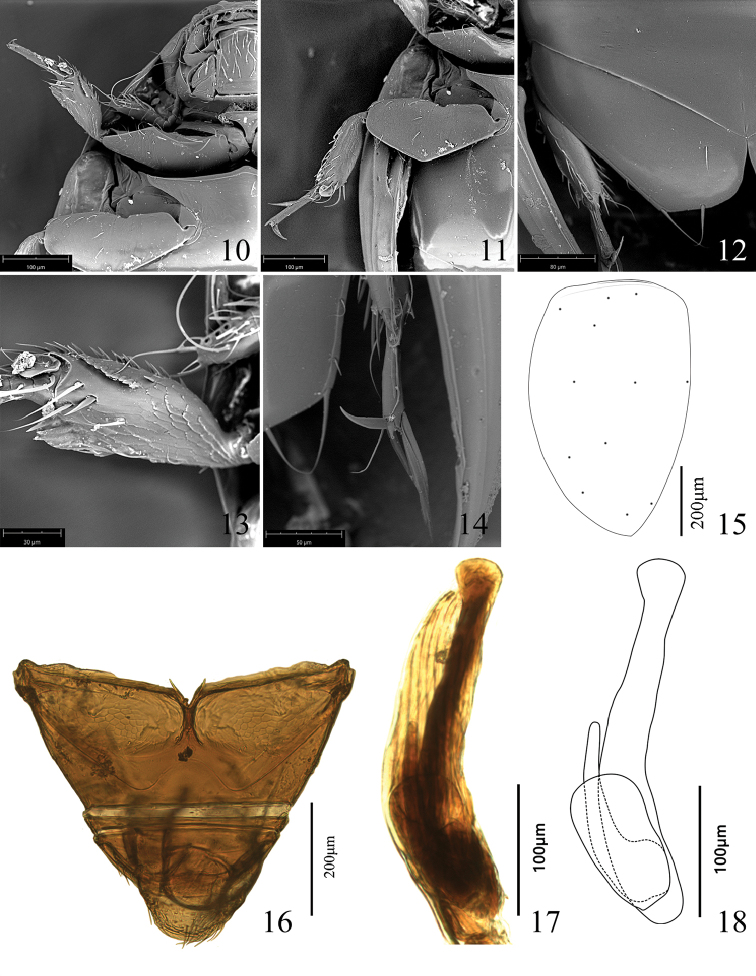
*Sphaeriusminutus* sp. n. **10** front leg **11** mid leg **12** hind leg **13** protibia **14** metatarsus **15** asperities on left elytron **16** abdomen **17** lateral view of male genitalia **18** illustration of male genitalia (lateral view).

*Legs.* Coxae transverse, procoxae close to each other, mesocoxae widely separated by meso-metaventrite, while metacoxae contiguous, forming a large transverse coxal plate with two setae at outer apical angle and middle of the posterior margin (Fig. 12). Profemur robust and broad, basal half of anterior margin rather concave, forming a cavity for head; mesofemur strongly dilated, short and broad, posterior side margin straight, forming obtuse angle; metafemur hidden under coxal plate. Protibia short and broad, bearing a row of short setae at inner edge and a row of denticles near the apex (Fig. 13); pro- and mesotibiae both bearing some long setae and a ventral disc at the apex (Figs 10, 11). Tarsus 3-segmented, first and second tarsomeres very short, shorter than wide, third tarsomere much longer, more than 3/4 as long as the entire tarsus; some setae on tarsus flattened and broadened, transforming into banding-shaped (Fig. 14). Claws quite well developed, strongly unequal, the smaller one ca. 0.5× as long as the other one in front legs, and much smaller in mid and hind legs (Figs 10, 11, 14).

*Abdomen.* Abdomen with three visible ventrites; first ventrite large and broad with two large coxal cavities occupying almost the entire ventrite, the basal half was divided into two parts by a longitudinal furrow in the middle; third ventrite shorter than first ventrite; second ventrite extremely narrow, ca. 0.15× as long as the third ventrite, anterior and posterior margins straight and parallel (Fig. 16).

*Male genitalia*. Median lobe long and laterally compressed, gradually narrowed toward apex, apex distinctly dilated, with apical angle rounded; left paramere digitus-shaped, half the length of the median lobe; right paramere petaliform (Figs 17, 18).

#### Etymology.

The species name comes from the Latin adjective *minutus* (= tiny), referring to the minute body size of this species.

#### Distribution.

Only known from type locality in Jiangxi Province, China.

#### Biology.

This species was found under a stone on the muddy shore of a running stream. They were tightly attached to the lower surface of the stone when the stone was removed. Biology of larvae unknown.

#### Differential diagnosis.

The new species closely resembles *S.papulosus* Lense, 1940 described from Myanmar. These two species share several character states. Besides the similar body shape and size, their antennal clubs are composed of four antennomeres, they both have smooth and shiny dorsal surface and with similar reticulation on pronotum, and they both have some conical and rough asperities on the elytra. However, the new species has a different pattern in the arrangement of the asperities and the metatarsus is 3-segmented instead of 2-segmented.

The new species differs from other species of *Sphaerius* by its less dilated antennal club. Additionally, the smooth, uniformly brown dorsal surface, the T–shaped prosternal process, the serial denticles on protibiae, and ventral disc on pro- and mesotibiae are also important characters to identify the new species. As for the median lobe, the Himalayan species have a more or less prominent apex, while the new species has a round and more dilated apex.

## Discussion

Based on their size and small wings and the fact that there is no record of Sphaeriusidae having been collected with traps, we infer that their ability to migrate is weak. All known species of *Sphaerius* in the Oriental Region occur in Nepal, Vietnam, and other areas of Southeast Asia (Lense 1936, 1940, Löbl 1995). The locality of the new species is rather distant from those of known species which suggests, besides the morphology, that we are dealing with an undescribed species.

Sphaeriusidae are frequently overlooked by collectors because of their minute size and special habitat environment. So, we believe that with proper collecting methods in suitable areas, more species would be discovered. The Oriental Region is obviously the hot spot of Sphaeriusidae, and all the Oriental species were described from countries bordering China. Therefore, we strongly believe there are more species distributed in south China.

## Supplementary Material

XML Treatment for Sphaerius minutus
